# Theoretical frameworks shaping healthcare incident reporting and learning systems: a scoping review

**DOI:** 10.1136/bmjopen-2026-124497

**Published:** 2026-11-09

**Authors:** Alia Al Baharnah, Paul Bowie, Anas Sattar, Julie Ferguson, Rabab Alkutbe, Catherine A O’Donnell

**Affiliations:** 1 School of Health and Wellbeing, University of Glasgow, Glasgow, UK; 2 Saudi Patient Safety Center, Riyadh, Saudi Arabia; 3 NHS Education for Scotland, Edinburgh, UK; 4 School of Health, Sport and Bioscience, University of East London, London, UK; 5 Medicine, NHS Education for Scotland, Glasgow, UK; 6 General Practice and Primary Care, University of Glasgow, Glasgow, UK

**Keywords:** Adverse events, Patient Safety, Quality of Health Care

## Abstract

**Abstract:**

**Objectives:**

This scoping review (ScR) aimed to identify and synthesise theoretical frameworks (TFs), applied philosophies and models used for the design, implementation and evaluation of healthcare incident reporting and learning systems (HIRLs) stages to map current theoretical underpinnings and guide future practices.

**Design:**

Scoping review following Joanna Briggs Institute methodology, guided by Arksey and O’Malley’s five stage framework. This review was reported using the Preferred Reporting Items for Systematic Reviews and Meta-Analyses-ScR guidelines. The protocol was registered on the Open Science Framework (OSF) (https://doi.org/10.17605/OSF.IO/5UQPE).

**Data sources:**

The databases Ovid MEDLINE, Embase, CINAHL and PsycINFO were searched from January 2009 to March 2026; supplementary manual search Google Scholar was also conducted.

**Eligibility criteria:**

We included published studies and grey literature if they explicitly used TFs for HIRLs development or evaluation in healthcare, focused on sentinel events or other types of adverse event/critical incident, were situated in healthcare systems and published in English. Criteria were structured using the ECLIPSE framework.

**Data extraction and synthesis:**

Standard methods were used to screen identified studies using Rayyan software. Data extraction focused on the study characteristics, HIRLs focus and nature of the theoretical application. Extraction was conducted by one reviewer then discussed with the study team. Charted data were synthesised descriptively and systemically organised by the type of TF, philosophy and foundational models and which stages of HIRLs these were applied to (design, implementation and evaluation). One author was initially screened to remove duplicates based on titles. Subsequently, title and abstract screening was independently conducted by three authors. The Rayyan software was used for screening. three author AAB, JF and AS. The Rayyan software was used for screening. The papers were screened using the inclusion and exclusion criteria.

The data were extracted using an Excel worksheet developed by the authors. Information regarding the first author, year, setting, study design, participant type and number of participants was recorded. Information on the type of TFs used and its specific applications in the design, implementation and/or evaluation of HIRLs was also extracted.

The synthesis of the results focused on the identification and description of the TFs, applied philosophies and foundational models and their use in the design, implementation and evaluation of HIRLs. This was achieved by summarising the literature according to the types of TFs’ applied philosophies and foundational models and the specific stages at which they were used.

A typology was constructed based on the types of TFs’ applied philosophies and foundational models used, using the categories defined by Nilsen, the underpinning perspective of the theory (eg, organisational management, sociology, psychology), and the point of application in the design, implementation or evaluation of HIRLs. Data extraction and synthesis were conducted by AB and discussed with PB and CAO’D. Any inconsistencies were resolved and agreed on by the team.

**Results:**

Of the 8713 studies identified, 28 met the inclusion criteria. Eight studies specifically used TFs, applied philosophies and models to analyse the reported incident content. Only the Theory of Planned Behaviour, Reason’s Swiss-Cheese model, Human Factors Engineering and Theoretical Domains Framework were used in more than one study. Other organisational, implementation and sociological TFs were also identified.

All the identified the TFs, applied philosophies and foundational models were mapped Goode’s systems thinking framework. Frameworks were largely explanatory (10 studies) or evaluative (9 studies), with 17 studies achieving ‘full integration’. However, a critical ‘middle-stage gap’ was identified: although theory was applied during pre-design (7 studies) and evaluation (12 studies), it was virtually absent during technical specification, data protocol development and system testing.

**Conclusion:**

This review maps the theoretical underpinnings of HIRLs evidence base and identifies the need for a consistent lifecycle approach to theory across all design stages. The findings provide the potential, along with other existing empirical evidence, to inform ‘good practice’ guidance for policy bodies, such as the WHO, national policymakers, healthcare organisations, educators and clinicians, to standardise reporting system design, implementation and evaluation. Future work must bridge technical design gaps and diversify geographical and professional perspectives to ensure that HIRLs have greater engagement from multidisciplinary teams and are globally applicable.

**STRENGTHS AND LIMITATIONS OF THIS STUDY**

- A comprehensive literature review was undertaken, incorporating searches across several electronic databases, manual search using Google Scholar, grey literature sources and citation-tracking tools.
- The review adheres to methodologically rigorous methods guidelines established by the Joanna Briggs Institute and is structured according to the Arksey and O’Malley framework.
- Mapping included studies to Goode *et al*’s systems thinking framework identified which parts of healthcare incident reporting and learning systems development are underpinned by theory, and which parts are not.
- One limitation of this scoping review is that only articles published in English were included, so pertinent literature may have been missed.

## Introduction

 Healthcare incident reporting and learning systems (HIRLs) are an important part of organisational learning and quality improvement initiatives, supporting patient safety within healthcare settings.^1^ HIRLs can potentially contribute to improved patient outcomes, and organisational learning and development.^2 3^ However, the design and implementation of HIRLs is complex, involves multiple stakeholders and is often implemented across institutional, regional and national levels.^4^ A range of reporting and learning approaches are employed within these systems. For example, root cause analysis (RCA) is commonly used in healthcare having been adapted from high-risk industries such as the aviation and oil and gas sectors.^5^ However, when applied in the healthcare system it reportedly showed limited consideration of the underlying principles and contextual requirement of the system complexities interactions influencing safety events. Peerally *et al* (2016) discuss eight challenges associated with the use of RCA in healthcare systems, highlighting the risk of investigations becoming a routine procedural exercise rather than opportunities for organisational learning. They also mention that oversimplification of event causes could lead to focusing on a single root cause, and highlighted the lack of expert investigators with appropriate training in human factors and system thinking. Other challenges include political and organisational pressures such as tight deadlines and insufficient autonomy of the investigators may lead to the prioritisation of completing the RCA report rather than facilitating organisational learning. It could also lead to ineffective corrective actions and weak interventions (such as reminder and training). Poor communication and feedback mechanisms are additional challenges leading to the reoccurrence of incidents. Moreover, investigating single incidents rather than investigating the whole system could also lead to reoccurring incidents and missing valuable learning opportunities and implementing effective corrective action. Unclear distinction between just culture, no blame approaches and accountability could lead to placing more pressure on the healthcare individuals involved, while providing minimum learning opportunities to help the system in preventing or minimising the risk of future incidents. Finally, fragmented governance and unclear responsibility for implementing risk mitigation action across organisation and system levels could hinder taking action.^5^ Peerally *et al* focused on RCA challenges during the technical design stages. Therefore, it is essential to adopt a holistic approach throughout all phases of designing such reporting and learning systems. Goode *et al*^6^ introduced a process framework for constructing incident reporting systems based on the principles of systems thinking and good practices for incident data collection, organisational learning, ongoing implementation and evaluation and system refinement and enhancement (figure 1).

**Figure 1:**
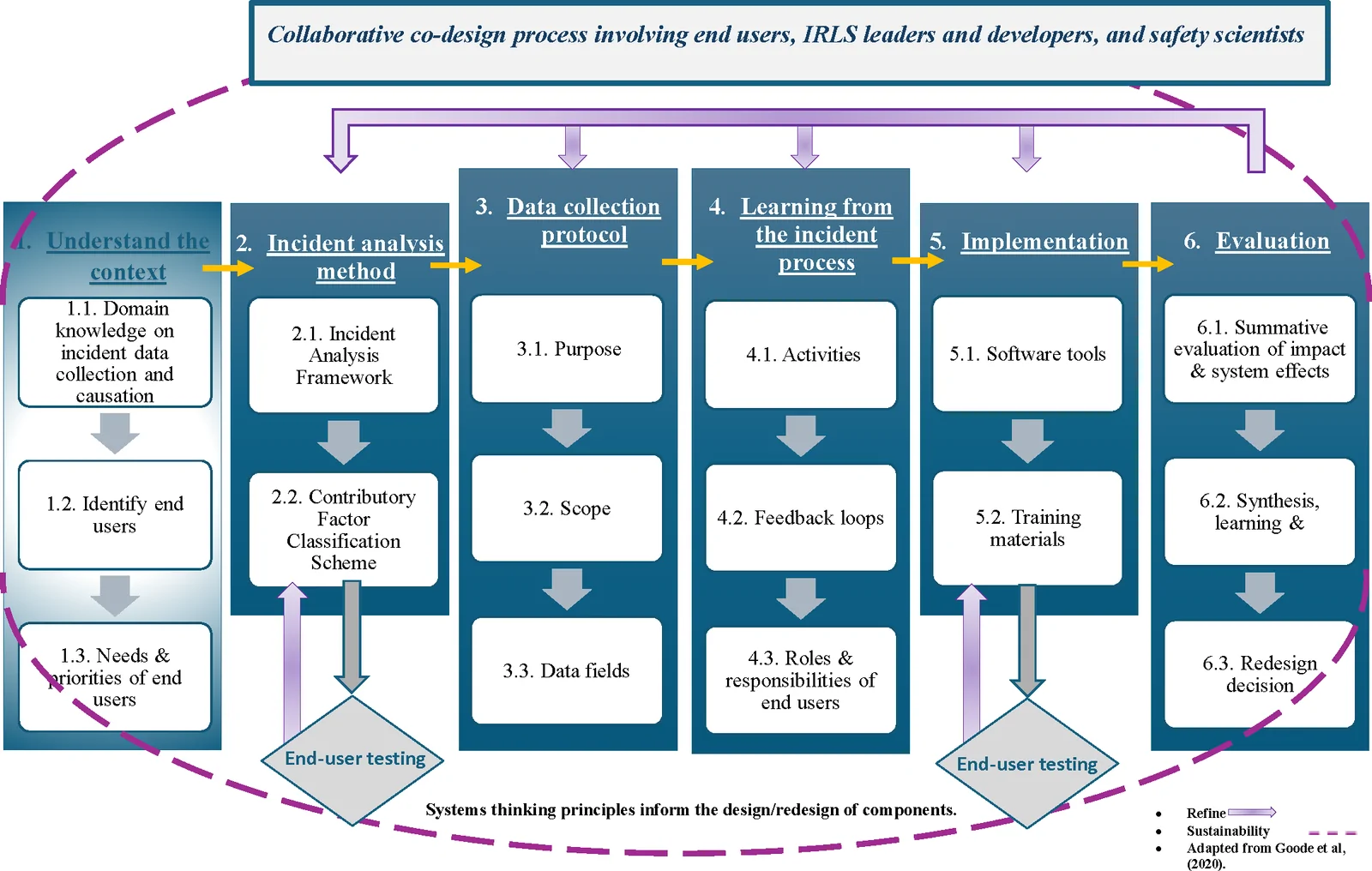
Systems thinking in the design and re-design of healthcare incident reporting and learning systems: a conceptual process model (adapted from Goode *et al*^6^).

Others have considered the challenges of using such systems. For example, Archer *et al* focused on identifying barriers to and facilitators of incident reporting to guide good practice.^7^ While fear of adverse consequences following incident reporting was the most commonly cited barrier, the second most common barrier was the process and systems of reporting. However, the process and systems of reporting were also identified as a major facilitator of reporting. It is due to such paradoxes (the same factor being both a barrier and an enabler) that led Kislov to call for clearer use of theory in the design and evaluation of quality improvement interventions such as HIRLs and to ensure that implementation strategies are evidence-based and contextually appropriate.^8^

However, although these studies developed frameworks of factors that may help facilitate or inhibit incident reporting system success, they did not address the role of theoretical frameworks (TFs), applied philosophies or models in underpinning the design, implementation or evaluation of HIRLs. This represents an important gap in our understanding of how HIRLs can and should function successfully. TFs, applied philosophies and models can guide HIRL development and implementation, and help us better understand how such systems are implemented and operate within highly complex healthcare contexts.^7–9^ For example, theory can help describe or guide implementation by making us more aware of who needs to be involved, explicate the resources required including finances and personnel, clarify what influences implementation outcomes, or aid the evaluation of implementation to determine what works (or not) and how it works.^10 11^

TFs, applied philosophies and models come from a range of research disciplines including management and organisational design, sociotechnical systems, human factors and sociology.^12^ This plurality of disciplinary approaches can offer invaluable insights into the structural, cultural and procedural conditions and interactions within healthcare organisations that influence implementation success.^13–16^ However, underpinning many of these theories, philosophies and models is the aim to identify and address what causes adverse or sentinel events. Put simply, what causes accidents when people interact with tools and technologies.^12^ Starting with a focus on root causes for error within systems, the recognition of a role for human factors and, latterly, a wider safety culture and climate has led to the development of overlapping theories and models such as Human Factors and Ergonomics (HFE) theory, Systems Theory and the Swiss Cheese model. Thus, sociotechnical theory explains that HIRLs can succeed only when technical tools and social environment—including people, workflows, culture and regulations—are designed and aligned together. It shows that failures in reporting systems usually stem not from technology alone but from mismatches among human, organisational and technical components.^17 18^

Despite advances in the development of HIRLs, a conspicuous gap remains in the literature regarding how TFs, applied philosophies and models underpin their design, implementation and overall functioning. A clearer understanding of which theoretical approaches are used, and at what stage of the HIRL design process would help to elucidate which parts of the HIRL implementation are well understood and which parts are not. For example, do we need to better understand the needs and priorities of users, better understand the structure of the data collected or more carefully consider evaluation of system performance. One challenge is the lack of clarity and agreement about what is meant by theories, philosophies and models.

Theory is defined as ‘an organized, heuristic, coherent and systematic articulation of a set of statements related to significant questions, providing a generalizable form of understanding’.^19^ Theory can operate at three levels: grand theory is abstract and applicable across areas and subjects.^11 20–22^ Mid-range theory addresses specific phenomena and concepts and lends itself to testable propositions or questions. Theoretical models are more narrowly defined and often only descriptive, although the difference between a model and a theory is not always apparent.^11^

In this work, we decided to embrace this conceptual fuzziness and, instead, focus on identifying which theoretical approaches or models have been reported in the literature in relation to the design and implementation of HIRLs and to identify where in the design and implementation of HIRLs theory has been applied.

This scoping review aimed to fill these gaps by mapping the theoretical foundations that shape the design and implementation of HIRLs, ultimately contributing valuable insights that can lead to improved patient outcomes and enhanced organisational performance.

The primary aim of this scoping review was to identify and explore the TFs, applied philosophies and foundational models that influence the design, functioning, implementation and evaluation of HIRLs. The objectives were to:

- Identify and outline the TFs, applied philosophies and foundational models used in the design, functioning, implementation and evaluation of included studies.
- Examine at what stages (eg, design, implementation, reporting, evaluation) these frameworks have been applied and provide the rationale for their use in each context.

## Methods

A scoping review of peer-reviewed empirical and theoretical literature was conducted following the Arksey and O’Malley framework^23^ and the methodological manual for scoping reviews released by Peters *et al*.^24^ A scoping review was selected owing to the broad and exploratory nature of the research method.

The review is reported in accordance with Preferred Reporting Items for Systematic Reviews and Meta-Analyses extension for Scoping Reviews (PRISMA-ScR): checklist and explanation to ensure comprehensive and transparent reporting.^25^ A completed PRISMA-ScR checklist for this scoping review is available in (online supplemental appendix 1). The scoping review protocol was registered in the Open Science Framework (OSF) (https://doi.org/10.17605/OSF.IO/5UQPE), and the protocol was published.^26^

### Search strategies

The ECLIPSE framework informed the development of the search strategies and inclusion and exclusion criteria (see online supplemental appendix 2). ECLIPSE stands for Expectation, Client Group, Location, Impact, Professionals and Service.^27^

In collaboration with an experienced medical research librarian, we developed a comprehensive search strategy based on the ECLIPSE criteria. The Ovid MEDLINE, Embase, PsycINFO and CINAHL databases were searched using the thesauruses specific to each resource. Additionally, supplementary manual searches were performed in Google Scholar using the keywords and phrases as shown in online supplemental appendix 3. The first 10 pages of the retrieved articles were checked for potentially missed references by the above-mentioned data bases.

Grey literature, including dissertations, conference papers, proceedings and reports, published on relevant websites, such as the WHO, Agency for Healthcare Research and Quality and Institute for Healthcare Improvement, were also searched. Searches were conducted on published papers between January 2009 and March 2026. Search Strings were categorised by thematic relevance, focusing on TFs or theory (String A), patient safety events (String B) and incident reporting systems (String C). To ensure comprehensive coverage, we do not use proximity operators in refining our search. The full search strategy is available in online supplemental appendix 3.

### Screening and data extraction

Papers identified through the search were imported into EndNote (V.21), and duplicates were removed. The retrieved records were initially screened by AAB to remove duplicates based on their titles. Subsequently, title and abstract screening was independently conducted by AAB, JF and AS. The Rayyan software was used for screening. The papers were screened using the inclusion and exclusion criteria (table 1).

**Table 1:** Inclusion and exclusion criteria

Inclusion criteria	Exclusion criteria
Papers focused on: Sentinel events, patient safety incidents, never events, adverse events, errors, critical incidents, significant events or near-miss events. Incident reporting systems at healthcare systems (HIRLs), healthcare, hospitals, primary care, secondary healthcare, ambulatory care, clinics, laboratory, pharmacy and dental clinics. Theoretical frameworks related to the design, implementation and evaluation of HIRLs stages to map current theoretical underpinnings and guide future practices. Interdisciplinary scientific disciplines, as well as applied philosophies and foundational models. English studies only. Healthcare incident reporting and learning systems.	Studies outside the specified date range. Incident reporting systems in other areas, eg, aviation, oil and gas industries. Any language other than English. Studies with no outcome about incident reporting. Papers with no empirical data.

HIRLs, healthcare incident reporting and learning systems.

The screening agreement was tested after the first 50 records to ensure a common understanding of the studies that fit the scope of the review. Frequent meetings were held to discuss uncertainties with the screening team. In cases of disagreement regarding inclusion, a consensus was reached through discussion or reference to a fourth reviewer RA. Records that passed the initial screening were then read in full text by AAB. Backward citation checking was performed on the reference lists of included studies and relevant reviews. To ensure the accuracy and relevance of the findings, results were reviewed by PB and CAO’D to ensure alignment with the overarching aims of the study. Database searches were supplemented by a search of Google Scholar, using keywords as shown in online supplemental appendix 3. The first 10 pages, ranked by relevance, were screened by AAB. This identified four additional peer reviewed papers, which were assessed against the same eligibility criteria and screening process as database records.

### Data extraction and synthesis

The data were extracted using an Excel worksheet developed by the authors. Information regarding the first author, year, setting, study design, participant type and number of participants was recorded. Information on the type of the TFs applied philosophies and foundational models used and its specific applications in the design, implementation and/or evaluation of HIRLs was also extracted.

The synthesis of the results focused on the identification and description of the TFs, applied philosophies and foundational models and their use in the design, implementation and evaluation of HIRLs. This was achieved by summarising the literature according to the types of TFs’ applied philosophies and foundational models and the specific stages at which they were used.

A typology was constructed based on the types of TFs’ applied philosophies and foundational models used, using the categories defined by Nilsen, the underpinning perspective of the theory (eg, organisational management, sociology, psychology),^11^ and the point of application in the design, implementation or evaluation of HIRLs. Each study was categorised according to level of TFs integration using a scale adapted from Trifiletti *et al* which assesses explicitness and consistency of a TF application throughout the research process.^28^ Four levels (L1-L4) of integration were defined; L1 (superficial): The theory is named but not clearly linked to the research questions, variables or methods. L2 (partial): Theory informs some elements but is not consistently applied in design, data collection and analysis. L3 (substantial): Theoretical constructs inform the selection and definition of variables and primary analytical strategy but are not comprehensively used to structure all findings. L4 (full): Theory shapes the problem statement, research questions, design, measures and analytical model and is explicitly used to interpret results and discuss how the study extends or refines the theory. Each theory was classified by its disciplinary origin, determined from the sources cited in the included studies (eg, behavioural, organisational, sociological, psychological or implementation science). Data extraction and synthesis were conducted by AAB and discussed with PB and CAO’D. Any inconsistencies were resolved and agreed on by the team.

### Risk-of-bias assessment or quality appraisal

Considering that this scoping review was designed to map the existing body of evidence, the authors did not assess the risk of bias or evaluate the quality of the included studies. This approach followed the methodology outlined in the Joanna Briggs Institute (JBI) methods^24^ manual and was consistent with established practices for scoping reviews of health-related subjects.

### Results

Searches identified 8713 papers; screening identified 24 articles which met the inclusion criteria, with an additional four studies identified and included manually (figure 2) and see online supplemental appendix 4 for the included studies.

**Figure 2:**
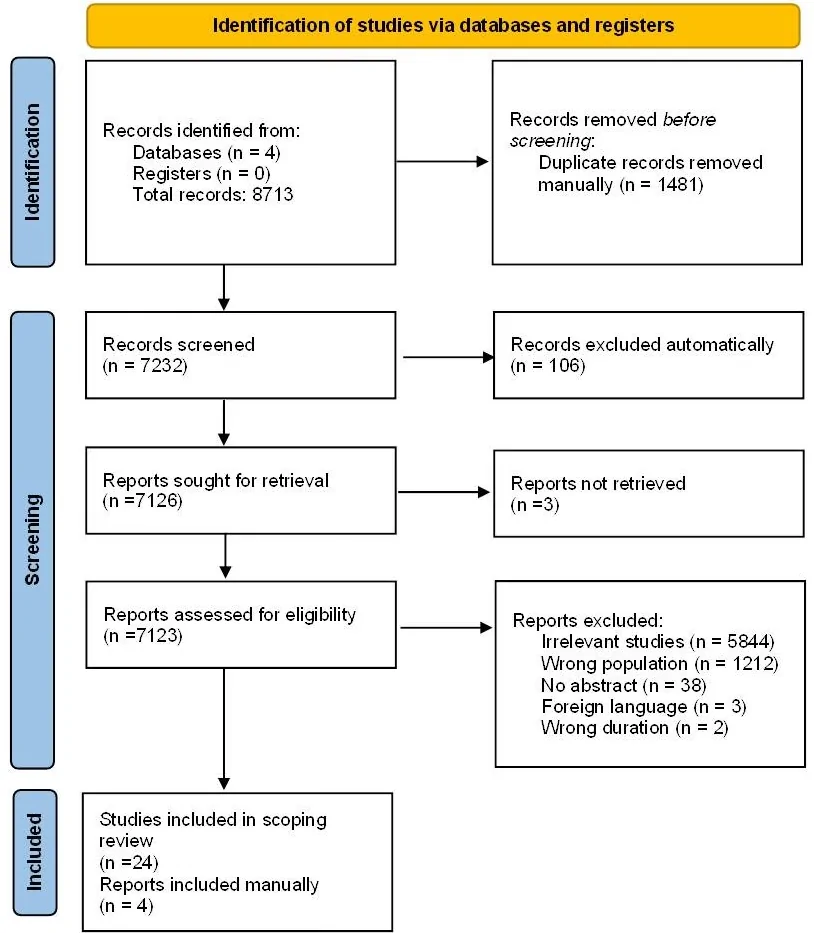
PRISMA-ScR checklist for a scoping review. PRISMA-ScR, Preferred Reporting Items for Systematic Reviews and Meta-Analyses extension for Scoping Reviews.

Studies were conducted in a range of country settings: seven in the USA,^29–35^ five in the UK,^7 36–39^ two each in Canada^40 41^ and Taiwan,^42 43^ and one each in the Netherlands,^44^ the Kingdom of Saudi Arabia,^45^ Qatar,^46^ UAE,^47^ Iran,^48^ Italy,^49^ Sweden^50^ and Spain.^51^ Four studies were conducted in multiple countries.^52–55^

Papers reported HIRLs at different levels: some focused on the reporter, others on incident reports and a small number on the reporting system itself. Approximately 19 studies reported multiple users of HIRLs.^7 29 32 33 36 39–48 53–55^ Overall, pharmacists were the most frequently reporting user group, which appeared in 14 studies,^7 32 33 36 39 40 42 43 46–48 53–55^ followed by nurses (12 studies)^7 29 33 36 40 42 43 46–48 54 55^ and by doctors (11 studies).^7 32 33 36 40 42 43 46 47 54 55^ Other groups included students, interns, dentists, dental assistants, administrative staff and healthcare professionals, incident investigators, quality managers, chairs of investigation committees, healthcare inspectors and other stakeholders (nine studies).^7 37 40–45 54 55^ Regarding incident reports, eight studies^30 31 35 38 49–52^ contained information on the incident reports, describing how studies analysed the incidents to identify TFs in the design and evaluation of HIRLs. Only one study discussed the reporting system^34^ (online supplemental appendix 5).

### Use of frameworks applied philosophies and model

24 TFs, applied philosophies and models were identified (table 2). Only Theory of Planned Behaviour (TPB), Reason’s Swiss-Cheese model, Human Factors Engineering (HFE) and Theoretical Domains Framework (TDF) were used in more than one study (two studies). Other organisational, implementation and sociological TFs were also identified (table 2). Some were adaptations from existing frameworks or developed by including additional concepts, such as modifying the Unified Theory of Acceptance and Use of Technology by integrating the Value of Perceived Consequence.^43^ Others described the underlying theory in vague terms, for example, the conceptual framework of social and participative learning and incident investigation processes.

**Table 2:** Type of framework, applied philosophies and model as described in the included studies

Theoretical framework type	Classification code	Number of included studies	Study citation
Theory of Planned Behaviour (TPB)	A	2	39 48
Theoretical Domains Framework (TDF)	A	2	46 47
Reason Swiss-Cheese model	B	2	49 54
Human Factors Engineering (HFE)	B	2	36 52
Human Factors Engineering (HFE) and Consolidated Framework for Implementation Research (CFIR)	H (B and E)	1	29
Human Factors Engineering (HFE) and ontology-based system design	H (B and D)	1	30
Human Factors Engineering (HFE) and a systems approach	B	1	53
Systems Engineering Initiative for Patient Safety (SEIPS) model	B	1	33
Mindset Theory	A	1	32
Social and Participative Learning Theory	C	1	44
Framework derived using a grounded theory approach	M	1	7
Unified Theory of Acceptance and Use of Technology (UTAUT)	D	1	43
Health Information Technology Classification System (HIT-CS)	D	1	50
Persuasive System Design (PSD) taxonomy	D	1	41
The WHO Patient Safety Reporting System Framework	F	1	34
TRIZ-based model (Theory of Inventive Problem Solving)	G	1	42
Risk Resiliency Concept adapted from the aviation industry to develop the Harm Susceptibility Model (HSM)	B	1	38
K-nearest neighbour (KNN)	M	1	35
Safety Culture Theory	C	1	40
The conceptual framework for patient safety and incident reporting systems (IRSs)	F	1	51
Goals, Operators (GOMS) model, theory of Human-Computer Interaction (HCI) and Keystroke-Level Model (KLM)	D	1	31
Argyris and Schön’s theory of organisational learning	C	1	55
Systems thinking theoretical framework, operationalised by the Contributory and Mitigations Factors Classification	B	1	37
Technology Acceptance Model (TAM)	D	1	45

Classification of theories: **Code (A):** Behavioural and psychological, **Code (B):** Human factors and systems safety (including accident causation), **Code (C):** Organisational and learning, **Code (D):** Technology acceptance, HCI and health informatics, **Code (E):** Implementation science, **Code (F):** Policy and guidance frameworks, **Code (G**)**:** Engineering design and problem-solving, **Code (H):** Hybrid (two foundations combined), **Code (M**)**:** Methodological, algorithm or analytical tool.

Across studies, the theoretical use considerably varied in terms of depth, purpose and methodological application. Some frameworks were primarily used to guide reporting system design or shape data collection, whereas others supported the analysis, interpretation or evaluation of learning and behavioural outcomes. A few studies used multiple overlapping theories, underscoring the inherent complexity of healthcare systems and the multifaceted nature of incident reporting and learning systems (table 2).

The analysis of TFs, applied philosophies and models across the collected studies revealed a diverse set of intellectual tools used to investigate specific domains or to suggest perspectives taken by the study team. This could highlight the interdisciplinary nature of the field. In total, 28 distinct or combined theoretical approaches were identified, demonstrating the broad theoretical foundation underpinning the research efforts (table 2).

The framework categories provided a functional classification of the 28 TFs, applied philosophies and models analysed, summarising their primary purpose within the research (table 3). This demonstrates that, in most included studies, frameworks were primarily used as explanatory or evaluative tools to help researchers understand the underlying reasons for and mechanisms of observed events (see online supplemental appendix 6).

**Table 3:** Mapping theoretical frameworks according to category, integration level and application stage in included studies

	Number of included studies	Study citation
(A) Framework categories		
Explanatory	10	7 29 32 36 39 40 46 47 49 53
Evaluative	9	30 34 38 41 42 44 50 51 55
Predictive	3	35 43 45
Descriptive and explanatory	2	33 54
Predictive and evaluative	2	31 48
Descriptive	1	37
Descriptive and evaluative	1	52
(B) Level of integration		
Full	17	32 33 35 36 38–40 42–44 46 49 50 52–54
Partial	10	7 29–31 34 41 47 48 51 55
Superficial	1	45
Substantial	0	^–^
(C) Application stage		
Evaluation	12	29–31 33 34 36 38 41 43 48 50 55
Design	7	32 39 45–47 53 54
Multistage (design, implementation and evaluation stages)	3	42 49 52
Multistage (design and evaluation stages)	2	37 40
Not specified	1	51
Implementation	1	35
Multistage (design and implementation stages)	1	7
Multistage (design, implementation, monitoring and evaluation stages)	1	44

Footnote:** (A):** The framework categories provided a functional classification of the TFs, applied philosophies and model analysis, summarising their primary purpose within the research **Explanatory: **(illustrate how or why incidents occur or/and reporting and learning happen), **Evaluative: **(assessing system performance based on well define criteria), **Predictive: **(prediction of outcomes, for example, reporting and learning intention or/and behaviour). **(B): **To examine to which degree the TFs, philosophies and models shaped the study **Full:** (theory shapes the problem statement, research questions, design, measures and analytical model and is explicitly used to interpret results and discuss how the study extends or refines the theory), **Partial:** (theory informs some elements but is not consistently applied in design, data collection and analysis) **Superficial: **(the theory is named but not clearly linked to the research questions, variables or methods), **Substantial: **(theoretical constructs inform the selection and definition of variables and primary analytic strategy, but are not comprehensively used to structure all findings). **(C): **The stage of HIRLs at which the TFs, philosophies and models was applied: **Design** (development of the system), **Implementation: **(operating the system including reporting and learning behaviour) **Evaluation:**(assessment the system performance and/or learning outcome, **Monitoring: **(ongoing tracking of the system function) and the **Multistage:** (applied at more than one stage).

HIRLs, healthcare incident reporting and learning systems; TFs, theoretical frameworks.

Explanatory frameworks were used in 10 studies,^7 29 32 36 39 40 46 47 49 53^ highlighting the focus on articulating causal relationships and underlying mechanisms. Nine studies^30 34 38 41 42 44 50 51 55^ used evaluative frameworks with a focus on assessing the efficacy, quality or impact of interventions and systems. Predictive frameworks that forecast future outcomes or behaviours were applied in three studies.^35 43 45^ The remaining studies used hybrid frameworks combining two or more functions (eg, descriptive and explanatory), demonstrating that many researchers use models with multiple, integrated objectives. The data indicate that the primary purpose of the TFs in the included studies is to either explain the process or evaluate systems/interventions, and a smaller proportion of frameworks were used to predict future outcomes or behaviours, as summarised in table 3.

This distribution highlights that the overarching goal of this study is primarily to understand and assess the current state, with comparatively little attention given to future scalability and sustainability. Most studies focused on understanding and assessing the current use, but little attention was paid to future scalability and sustainability.

Of the 28 included studies, 17 were classified as full integration of TFs, philosophies or models. 10 as partial integration, 1 as superficial integration and no study was classified as substantial (table 3).

The included studies were reviewed to identify the specific stages (design, implementation, monitoring and evaluation) at which the TFs, applied philosophies and models were applied. Full details are provided in online supplemental appendix 3. Most studies (12 in total) applied a TF at the evaluation stage, whereas seven applied a framework at the design stage. Other studies applied TFs, applied philosophies and models across multiple stages. One study did not identify the stage at which the framework was used. Finally, six studies applied a framework to the implementation stage of HIRL, although this was often part of a multistage process (table 3).

Figure 3 presents the distribution of the identified TFs, applied philosophies and models, illustrating where they were applied and outlining the rationale for their use across the stages of a design or redesign process, as adopted from Goode *et al.*^6^ This shows that the application of TFs, applied philosophies and models occurs mainly in stages 1 (pre-design), 5 (post-design evaluation) and 6 (post-implementation monitoring). A notable limitation identified in the literature is the scarcity of studies investigating or applying TFs, applied philosophies and models during stages 2 (design specification), 3 (design implementation) and 4 (design testing). This pattern suggests that TFs, applied philosophies and models are predominantly applied during the pre-system and post-system design phases, with emphasis placed on the initial development or subsequent assessment of HIRLs rather than on the iterative stages of system development and testing (figure 3).

**Figure 3:**
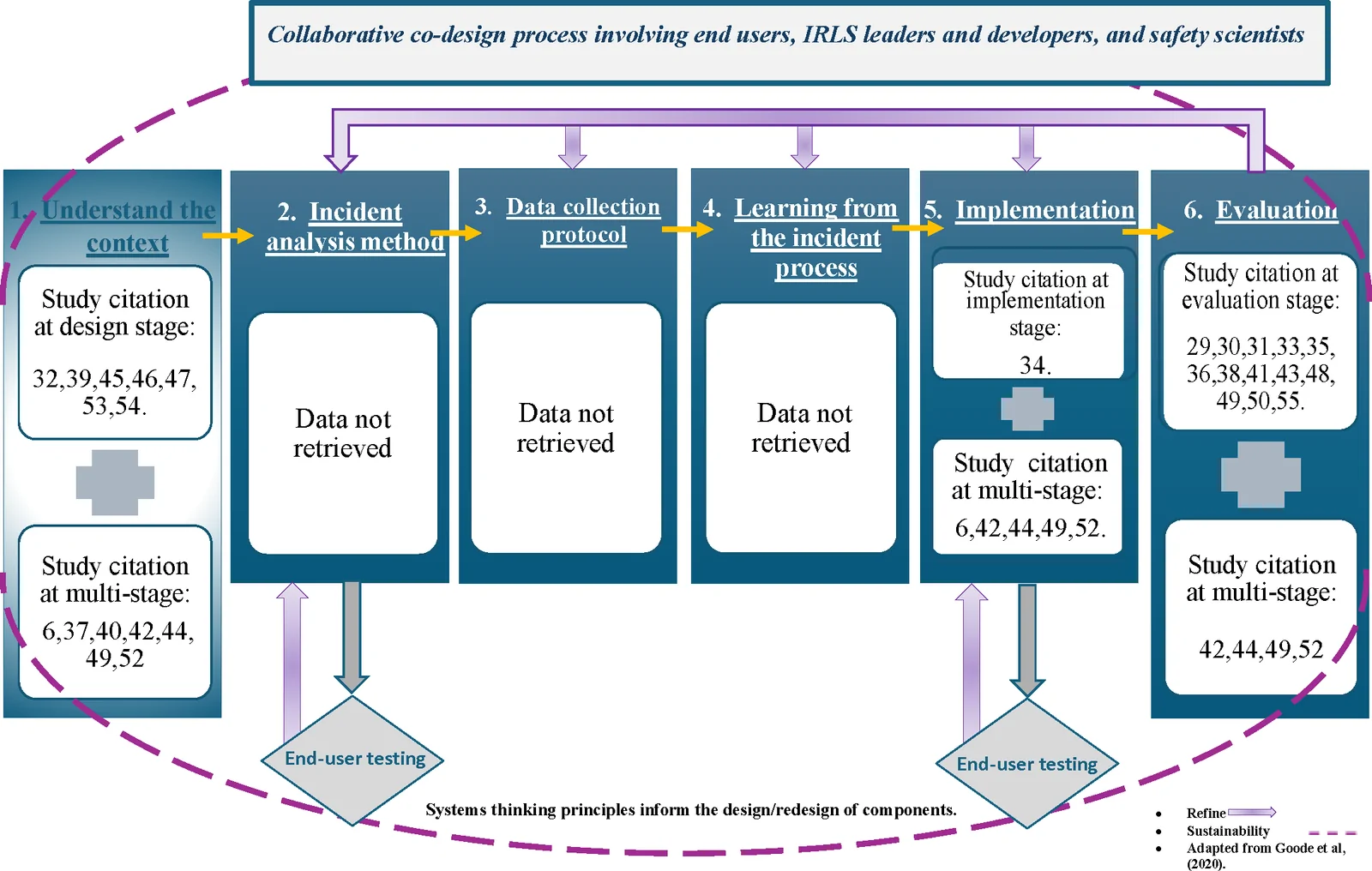
Framework types used throughout the design and redesign phases of HIRLs (adapted from Goode *et al* 2020). HIRLs, healthcare incident reporting and learning systems; IRLS, incident reporting and learning systems.

Table 3 provides a concise analysis of the functional placement of the 28 identified TFs, applied philosophies and models within the design and redesign processes across the reviewed literature. The data clearly indicate a significant research bias towards post-design assessment, with the evaluation stage accounting for the largest share of applications in 12 instances (42.9%). This strong focus on evaluation suggests that TFs, applied philosophies and models are most frequently used for retrospective assessments and critique to measure the usability or impact of an implemented system or initial HIRLs.

In contrast, the design stage, in which TFs, applied philosophies and models are typically used to inform and build the initial conceptual blueprint and requirements, accounts for the next largest category in seven instances (25.0%). This is followed by various multistage applications that show less frequent but more comprehensive use across different phases. For instance, multistage (design, implementation and evaluation stages) comprises three instances (10.7%), demonstrating that few studies track a framework’s influence across the full system lifecycle. Crucially, the implementation stage, in which the design is actually executed, is rarely the primary focus, appearing only once (3.6%). This distribution underscores a critical finding: TFs, applied philosophies and models in the analysed studies are primarily used as tools for understanding and judging outcomes, rather than being consistently integrated as guidance for iterative development and real-time execution (table 3).

In figure 3, the framework types used throughout the design and redesign phases of HIRLs presents a visual analysis of how the identified TFs, applied philosophies and models are distributed across the six stages of the generic design and redesign process.

## Discussion

This scoping review aimed to identify, explore and map the TFs, applied philosophies and models underpinning HIRLs, the stages at which they were applied and the rationale for their use. Analysis of the 28 included studies identified a range of TFs, with behavioural theories, such as the TPB and TDF. Overall, the findings demonstrate that the field is influenced by behavioural psychology and systems engineering; however, it remains constrained by a critical and persistent ‘hourglass’ or ‘phase gap’, wherein theoretical application largely disappears during the core technical stages of system development. All included studies were mapped to Goode *et al*’s systems thinking framework as shown in figure 3 which reveals a marked concentration of TF application at the early and late stages of the process, particularly during stages 1 (understanding the context) which involves the initial identification and conceptualisation of HIRLs and stage 6, which is the final assessment phase. Furthermore, stage 5 (implementation) has limited evidence of applications. This suggests that TFs, applied philosophies and models were most often applied for post-design assessments and impact measurements. However, there is a major gap in the core developmental phases, with stage 2 (incident analysis method), stage 3 (data collection protocol) and stage 4 (learning from the incident process) all marked as ‘Data not retrieved’. This finding indicates the limitation of research on the application of TFs, applied philosophies and models as prescriptive guides in technical design specifications, implementation planning and formal learning structures. This has led to create a gap between theoretical principles and technical and operational practice. It illustrates the importance of focusing on the technical stages (stage 2–4) as well as translating outcomes into learning opportunities through effective feedback and corrective actions.^5^ Within the reviewed literature, this distributional imbalance suggests that TFs, applied philosophies and models function primarily as diagnostic tools for initial problem understanding and final outcome judgement, rather than being consistently integrated as guiding tools throughout the iterative, practical system building and requirement testing that occurs in the middle stages of the design process.^5^ Despite the conceptual richness, critical structural weaknesses persist. This scoping review identified a pronounced ‘hourglass’ pattern of theoretical integration across the design lifecycle. This theoretical gap suggests that although system intent and outcome assessment are theoretically informed, the construction of system logic, data structures and testing protocols is frequently driven by pragmatic or ad hoc technical decisions. As Goode *et al* argued, the absence of a continuous theoretical thread undermines the alignment between identified user needs and the final technical artefact.^6^ The application stages and rationale illuminate this gap. During the design phase, frameworks, such as TDF, Technology Acceptance Model and systems thinking, were used to support user-centred design and enhance the usability, accessibility and appropriateness of reporting protocols and interfaces.^37 45^ The implementation phases benefited from the HFE and Consolidated Framework for Implementation Research (CFIR), which facilitated the understanding of contextual enablers and barriers.^29 42^ Behavioural theories further support the identification of social and psychological determinants that influence reporting behaviours.^47 48^ Evaluation and monitoring were most strongly represented by frameworks, such as Reason’s model, Systems Engineering Initiative for Patient Safety (SEIPS) and the Harm Susceptibility Model applied to assess reporting processes and risk patterns.^33 35 54^ Although these frameworks enhance retrospective analysis and learning, their dominant use in the evaluation stage reinforces the field’s reliance on explanatory rather than predictive approaches.

Behavioural theories were the most frequently represented theoretical approaches in HIRLs. In particular, the TPB and TDF confirm that incident reporting is primarily conceptualised as a human behavioural challenge rather than a purely sociotechnical one. Studies using these frameworks^46–48^ moved beyond interface-level concerns to interrogate socio-psychological determinants. This emphasis aligns with Trifiletti *et al*, who argued that effective interventions must be grounded in a theory capable of explaining the mechanisms of behavioural change.^28^ Importantly, these findings reinforce the notion that the intention to report does not automatically translate into actual reporting behaviour, highlighting the need for system-level enablers and sustained user engagement.^48^ Moreover, the application of HFE, systems thinking and the SEIPS model is consistent with the sociotechnical perspective on patient safety.^33 36^ These frameworks conceptualise safety and performance as emerging from interactions among person, task, physical environment, technology and tools and the organisation of work rather than just resulting from individual-level factors.^56 57^ Their application supports a more holistic approach to HIRL design and implementation, ensuring that requirements extend beyond just user motivation. This includes system usability, accessibility, task alignment and organisational contexts, for example, governance, feedback mechanisms, resources and healthcare employee capacity building to act on effective reporting incidents.^57^

In terms of level of integration, most of the included studies demonstrated the ‘Full Integration’ of TFs, applied philosophies and models indicating increasing methodological rigour and a move away from superficial theory application into HIRLs research.^58 59^ Hybrid approaches, such as combining HFE with CFIR,^29^ exemplified this maturation. However, 10 studies were classified as partially integrated and one study superficially integrated, highlighting the ongoing challenges in fully operationalising the theory. This observation aligned with concerns highlighted by Peerally *et al* regarding the implementation of patient safety tools without sufficient consideration of their underlying principles and contextual requirements.^5^

This limitation is compounded by extreme fragmentation across the evidence base. Majority frameworks (20 of 28 studies) were unique models that were applied only once, preventing the development of shared, validated analytical tools. This fragmentation directly undermines analytical capacity and standardisation. Explanatory frameworks were the most frequently used in 10 studies, whereas predictive frameworks remained scarce in three studies, reinforcing a retrospective orientation towards understanding past events rather than predicting risk. Consequently, the lack of robust data collection and analytical tools limits scalability and generalisability, particularly given the strong focus on the evaluation of 12 studies and localised design interventions in seven studies (table 3).

The strength of this scoping review includes the methods adhered via the rigorous guidance of the JBI and Arksey and O’Malley framework. A comprehensive search strategy encompassing academic databases, manual search using Google Scholar, grey literature and citation tracking was used. Although data extraction and synthesis were conducted by one reviewer (AAB), bias was reduced through regular debriefings with the research team (CAO’D and PB) and verification of a sample of extracted data. The theory underpinning each included study was identified and then mapped to Goode *et al*’s systems thinking framework and identified which parts of HIRLs development.

Nevertheless, this study has some limitations. The evidence base was geographically concentrated in the USA (25%) and the UK (17%), constraining global generalisability. Reporting cultures are deeply embedded in local, legal, professional and hierarchical contexts, meaning that Western models may not fully translate into other settings. In addition, reliance on academic publications may exclude internal healthcare industry design manuals and proprietary frameworks. The observed ‘middle-stage gap’ may also reflect reporting bias, whereby theoretical input during technical design was undertaken but insufficiently documented. Finally, the classification of integration levels inherently involves some subjectivity, despite the use of established criteria. In clinical practice, this review underscores the necessity of integrating multiple theoretical perspectives to address the psychological, organisational, technical and system-level determinants of incident reporting. The identified frameworks (see figure 3) directly support user-centred design, targeted training, cultural change, resource allocation and continuous feedback mechanisms, demonstrating tangible real-world utility.

Future studies should prioritise bridging the theoretical gap across the design and testing phases of HIRLs. Studies must explicitly document how the HFE, Cognitive Task Analysis and systems thinking approaches inform technical specifications and data collection protocols. Thus, there is a clear need to move from explanatory models towards predictive approaches. Integrating behavioural theory with quantitative and machine learning techniques, such as k-nearest neighbour algorithms^35^ offers a pathway for transforming incident reporting from a retrospective function into a proactive early warning system.

Another interpretation around the use of theory is that there is no real consistency or even comment on which theories are best suited to this topic. The theories identified are broad and diverse, almost as if there is a ‘wild west’ approach to considering which theoretical underpinnings are potentially useful in this domain. Future researchers should begin to critically explore the utility and usefulness of underpinning theory in the design, implementation and evaluation of HIRLs over time.

It is also equally important to expand stakeholder inclusivity. Although pharmacists and nurses comprised the primary participant group of studies, the development of genuinely ‘high-level’ requirements necessitates active engagement from physicians, managers and administrative leaders, and other stakeholders. Finally, extending the research to low- and middle-income countries is essential for adapting and validating frameworks across diverse cultural and organisational contexts.

## Conclusion

This scoping review provides a comprehensive and integrated map of the TFs, applied philosophies and models underlying the design, implementation and evaluation of HIRLs. Although the field demonstrates increasing methodological maturity and theoretical diversity, the persistent absence of theory during the core technical stages poses a substantial risk to system effectiveness. Addressing this ‘middle gap’ through lifecycle-based, predictive and context-sensitive theoretical integration is critical if HIRLs evolve from passive data repositories into dynamic engines of organisational learning, and sustained patient safety improvement.

## Supplementary material

10.1136/bmjopen-2026-124497 online supplemental file 1

## Data Availability

Data are available upon reasonable request. All data relevant to the study are included in the article or uploaded as supplementary information.
