# Supplementary material for: Theoretical frameworks shaping healthcare incident reporting and learning systems: a scoping review
Source: BMJ Open. 2026 Nov 9;16(11):e124497. doi: 10.1136/bmjopen-2026-124497 (PMC13756437; doi:10.1136/bmjopen-2026-124497)
Supplement: Supplementary file 1 [file bmjopen-2026-124497.reviewer_comments.pdf]

PEER REVIEW HISTORY

BMJ Open publishes all reviews undertaken for accepted manuscripts. Reviewers are asked to complete a checklist review form and are provided with free text boxes to elaborate on their assessment. These free text comments are reproduced below.

ARTICLE DETAILS

Title (Provisional)

Theoretical Frameworks Shaping Healthcare Incident Reporting and Learning Systems: A Scoping Review.

Authors

Al Baharnah, Alia; Bowie, Paul; Sattar, Anas; Ferguson, Julie; Alkutbe, Rabab; O'Donnell, Catherine A

VERSION 1 - REVIEW

**Reviewer**	**1**
**Name**	**Poots, Jill**
**Affiliation**	**Trinity College Dublin**
**Date**	**21-Jul-2026**
**COI**	**None**

This is a great paper and there is great need for it in the field. My only minor comments are around A) It would be great to have more of a discussion of why Learning Systems lose their grounding in evidence, B) the presentation of human factors as being separate to systems thinking and the Swiss-cheese model, C) the methods, D) careful reflection of the limitations, E) a minor comment about the presentation of grounded theory in the results and F) I think you are talking about implementation (science) without using the term implementation science!

A) Some of the 'Problem with' papers in the BMJ might be useful reading. e.g. Peerally, M. F., Carr, S., Waring, J., & Dixon-Woods, M. (2017). The problem with root cause analysis. BMJ quality & safety, 26(5), 417-422.

B) In your introduction you situate all the possible theories, which is interesting, but I can't help but think it might be better 'anchored' to accident causation theory, as this is ultimately what you are describing. You pitch HFE as if it is completely distinct from systems thinking or Swiss-cheese when the practical reality is there is much crossover. Rewriting a couple of sections to be about accident causation might help improve this. You might also find Waterson et al's paper on the history of STS useful, to see where the overlap starts: https://www.tandfonline.com/doi/full/10.1080/00140139.2015.1015622#d1e238

C) There is a bit of conflict in the methods as you describe multiple screeners, but then in your limitations you describe only one screener. Please clarify which stages were carried out by whom. I would also like more description of the process for clarification and consensus, which you deem to be important in the limitations.

D) The limitations section spends more time discussing the limitations of the literature base than the limitations of the authors and this paper which is common but demonstrates little reflection! Please consider the limitations of this review and present them more transparently. For example, you describe one screener - this is a limitation but you go on to write that it doesn't really matter anyway... which isn't really a reflective limitation. You might consider more concrete limitations such as the use of a scoping review vs a systematic review, the lack of evidence quality/risk of bias. It doesn't tarnish the excellent piece of work that you have done, but rather provides evidence of reflection, learning and sets examples for others who can pick up your work and continue to advance this field.

E) Very minor but I have always known grounded theory to be more methodological and isn't a framework as such that is comparable to the others you have explicitly named, so I was surprised to see it in your table. I might suggest you rephrase to 'framework derived using a grounded theory approach' if this is what you mean.

F) Regarding your thoughtful description of teh phases of implementation - it might strenghten this article to refer to an implementation science/knowledge translation framework

I'll finish by saying I thought this was well-written.

**Reviewer**	**2**
**Name**	**Peddle, Monica**
**Affiliation**	**Deakin University**
**Date**	**13-Oct-2026**
**COI**	**None**

Thank you for the opportunity to review your scoping review. Detailed comments and feedback are provided in the attached PDF for your consideration in an effort to improve the reporting in a revised version.

**** The reviewer provided a marked copy with additional comments. Please contact the publisher for full details. ****

VERSION 1 - AUTHOR RESPONSE

Comments from Reviewer	Comments Page Number	Reviewers Comments	Authers response
**Reviewer 1**		Comments to the Author:<br>This is a great paper and there is great need for it in the field. My only minor comments are around:<br><br>A) It would be great to have more of a discussion of why Learning Systems lose their grounding in evidence,<br>B) the presentation of human factors as being separate to systems thinking and the Swiss-cheese model,<br>C) the methods,<br>D) careful reflection of the limitations,<br>E) a minor comment about the presentation of grounded theory in the results and<br>F) I think you are talking about implementation (science) without using the term implementation science!	Thank you for your positive feedback. We have dealt with your comments (A) to (F) in turn, following your more detailed feedback below.
		A) It would be great to have more of a discussion of why Learning Systems lose their grounding in evidence.<br>Some of the 'Problem with' papers in the BMJ might be useful reading. e.g. Peerally, M. F., Carr, S., Waring, J., & Dixon-Woods, M. (2017). The problem with root cause analysis. BMJ quality & safety, 26(5), 417-422.	Thank you for this helpful comment and for sharing the suggested articles. We have drawn on Peerally et al. in the Introduction, where we outline the challenges that can cause learning systems to drift from their founding principles (page 5), and in the Discussion, where we relate this to our finding that theory is rarely applied at the analysis and learning stages of these systems (page 18). As the primary aim of this review was to identify and explore the theories shaping HIRLs, we have kept this discussion focused on its relevance to our findings.
		B) The presentation of human factors as being separate to systems thinking and the Swiss-cheese model. In your introduction you situate all the possible theories, which is interesting, but I can't help but think it might be better 'anchored' to accident causation theory, as this is ultimately what you are describing. You pitch HFE as if it is completely distinct from systems thinking or Swiss-cheese when the practical reality is there is much crossover. Rewriting a couple of sections to be about accident	We thank the reviewer for this comment. We have addressed this point on page 6 and 7.

		causation might help improve this. You might also find Waterson et al's paper on the history of STS useful, to see where the overlap starts: https://www.tandfonline.com/doi/full/10.1080/00140139.2015.1015622#d1e238	
		C) There is a bit of conflict in the methods as you describe multiple screeners, but then in your limitations you describe only one screener. Please clarify which stages were carried out by whom. I would also like more description of the process for clarification and consensus, which you deem to be important in the limitations.	We thank the reviewer for their comment. The screening process was conducted blindly by three reviewers and discrepancies were resolved by fourth reviewer. And then data extraction and synthesis were conducted by one reviewer and discussed with rest of authors. (Page 9 and 10)
		D) The limitations section spends more time discussing the limitations of the literature base than the limitations of the authors and this paper which is common but demonstrates little reflection! Please consider the limitations of this review and present them more transparently. For example, you describe one screener - this is a limitation, but you go on to write that it doesn't really matter anyway... which isn't really a reflective limitation. You might consider more concrete limitations such as the use of a scoping review vs a systematic review, the lack of evidence quality/risk of bias. It doesn't tarnish the excellent piece of work that you have done, but rather provides evidence of reflection, learning and sets examples for others who can pick up your work and continue to advance this field.	We thank the reviewer for this thoughtful comment and have rewritten the limitations section (page 21).
		E) A minor comment about the presentation of grounded theory in the results. I have always known grounded theory to be more methodological and isn't a framework as such that is comparable to the others you have explicitly named, so I was surprised to see it in your table. I might suggest you rephrase to 'framework derived using a grounded theory approach' if this is what you mean.	Thank you for this comment. We also agree that grounded theory is a methodological approach to qualitative research, rather than a framework. In Table 2, we sought to use the theory or framework description used by the authors themselves, rather than apply our own interpretation of what they meant. However, your suggestion is pragmatic and a good way to deal with this. We have, therefore, used your suggested description in Table 2.

		F) Regarding your thoughtful description of teh phases of implementation - it might strenghten this article to refer to an implementation science/knowledge translation framework<br><br>I'll finish by saying I thought this was well-written.	We thank the reviewer for this comment. We have addressed this point on page 7.
**Reviewer 2**	Page: 4	As the PRISMA-Scr are guideline for reporting not conducting the review please update this to read This review was reported using ...	We thank the reviewer for this valuable comment and have revised the text accordingly.
		There are a number of abbreviations in the abstract which does not follow the author guidelines please check	We thank the reviewer for this valuable comment and have revised the text accordingly.
	Page: 7	There is no context provided for the studies cited to assess alignment or the context in which they were conducted such as what was studies and why it relevant here.	Article word limit prevented us from offering a detailed explanation of our choices of cited study here. The work of Goode et al is a well-recognised resource addressing how to design incident reporting systems. The work of Archer et al is important as they sought to identify the barriers and facilitators to *using* such systems, once implemented. In doing so, Archer et al identified the paradox of the same variable being regarded as both a major barrier and a major facilitator.<br><br>To make this clear, we have augmented our rationale on pages 6-7 as follows:<br><br>*Others have considered the challenges of using such systems. For example, Archer et al. focused on identifying barriers to and facilitators of incident reporting to guide good practice. While fear of adverse consequences following incident reporting was the most commonly cited barrier, the second most common barrier was the process and systems of reporting. However, the process and systems of reporting was also identified as a major*

			facilitator of reporting.[6] *It is due to such paradoxes (the same factor being both a barrier and an enabler) that led Kislov to call for clearer use of theory in the design and evaluation of quality improvement interventions such as HIRLs and to ensure that implementation strategies are evidence-based and contextually appropriate.*[7]
	Page: 8	This section describes a range of theories, frameworks and models; however, it does not provide a rationale for why their use is important in HIRL research. The authors should explain how these approaches add value, rather than simply listing examples.	Thank you for this. On page 7, we have expanded our text to further explain how theory might aid understanding of what contributes to successful implementation.<br>*.... help us better understand how such systems are implemented and operate within highly complex healthcare contexts.*[6-8] *For example, theory can help describe or guide implementation by making us more aware of who needs to be involved, explicate the resources required including finances and personnel, clarify what influences implementation outcomes, or aid the evaluation of implementation to determine what works (or not) and how it works.*[9,10]
		At present, the manuscript does not provide a clear rationale for the study. While the importance of theories, frameworks and models in HIRL is asserted, there is insufficient explanation of why they matter for research design, implementation, evaluation, or knowledge translation. The authors also state that the literature lacks robust exploration of TF use; however, no evidence is provided to support this claim. Establishing this gap is a key purpose of the background section and should be supported by relevant literature. The introduction would benefit from a stronger justification for the review, including evidence of the problem being addressed and the significance of understanding TF use within HIRL research.	We thank the reviewer for this comment this has been addressed.

		Why are there a series of single sentence paragraphs	This was a formatting error and has now been corrected.
		It would be helpful for the authors to clearly distinguish between theories, theoretical frameworks, philosophies, and foundational or conceptual models, as these are not synonymous and serve different purposes within research. At present, these concepts appear to be used interchangeably, which may create confusion for readers. Providing clear definitions and describing the distinct role of each would strengthen the conceptual clarity of the review and assist readers in interpreting the findings	We agree that it would be helpful clearly distinguish between theories, frameworks, philosophies and models. Unfortunately, the literature itself is unclear on these terms, and they are often used interchangeably. We have tried to address this by expanding our definitions on introduction page 6 and 7.
	Page: 9	it is reported using	Thank you for your comment, this has been updated "The review is reported in accordance with Preferred Reporting Items for Systematic Reviews and Meta-Analyses Extension for Scoping Reviews (PRISMA-ScR)"
		What is meant by Google Scholar keywords? It reads like Google Scholar has a set of key words like MESH headings. Please clarify.	Thank you for your comment, this has been revised as follows "Additionally, supplementary manual searches were performed in Google Scholar using the keywords and phrases as shown in appendix 3"<br><br>Moreover, appendix 3 has be updated and a section was added to include Google Scholar with following keywords "Incident reporting", "patient safety events""learning system", "theory", "framework"
		I would normally expect to see the key words or concepts searched along with key limiters in the paper this would help readers understand the review evidence base and also improve transparency.	We thank the reviewer for this helpful suggestion. We have now clarified the paragraph. "Search Strings were categorised by thematic relevance, focusing on theoretical frameworks or theory (String A), patient safety events (String B), and incident reporting systems (String C). To ensure comprehensive coverage, we

			don't not use proximity operators in refining our search. Presents the full search strategy is available (Appendix 3).
	Page: 10	1. by who? You have listed team members above but not here.<br><br>2. How did this happen what was the process needs to be transparent for replication	1. Thank you very much for your comments the authors' initials is now added.<br>2. Thank you for your observation, the paragraph has been updated accordingly "Database searches were supplemented by a search of Google Scholar, using keywords as shown in appendix 3. The first 10 pages, ranked by relevance, were screened by AB. This identified four additional peer reviewed papers, which were assessed against the same eligibility criteria and screening process as database records."
	Page: 11	So above it was TF applied philosophies and foundational models - why has it changed to just TF	We thank the reviewer for this comment. We have updated the sentence accordingly (the TFs applied philosophies and foundational models)
	Page: 12	this is awkward use of and	We thank the reviewer for this comment. We have revised the sentence and removed the repetition.
		So is these two studies used all these or was each listed reported in two studies this is not clear.	We thank the reviewer for this comment. The sentence has been revised for clarity as follows:<br><br>Twenty- four TFs applied philosophies and models were identified (Table 2). Only the Theory of Planned Behaviour (TPB), Reason's Swiss-Cheese model, Human Factors Engineering (HFE), and Theoretical Domains Framework (TDF) were used in

			more than one study. Other organisational, implementation, and sociological TFs were also identified.
	Page: 13	I am beginning to consider that some approaches may primarily inform the design of systems or interventions, whereas others are more relevant to implementation, evaluation, quality improvement, or the analysis of errors and outcomes. Recognising these differences may provide a more nuanced understanding of how and why particular approaches are used within HIRL research. Potentially a mapping of the approach with the use may be helpful to assess alignment between the approach and the use.	Thank you for your observation. Table 3 illustrates the mapping of framework types used throughout the design of HIRLs which is adapted from Goode et al., 2019. The table legend is now added.
		I am concerned that disproportionate emphasis is placed on approaches identified in two studies, while numerous other approaches identified in a single study I am concerned that disproportionate emphasis is placed on approaches identified in two studies, while numerous other approaches identified in a single study receive limited discussion. The difference is only 1 citation.	Thank you for your comment and we agree with these points. We have revised this section as the following:<br><br>The analysis of TFs applied philosophies and models across the collected studies revealed a diverse set of intellectual tools used to investigate specific domains or to suggest perspectives taken by the study team. This could highlight the interdisciplinary nature of field. In total, 28 distinct or combined theoretical approaches were identified, demonstrating the broad theoretical foundation underpinning the research efforts (Table 2) - alternatively, there appears to be limited theoretical consensus within the field.
		I do not agree with this contention. Given the minimal difference in frequency, the rationale for privileging certain approaches is unclear and may overstate their prominence within the literature. The authors should justify this approach or present the findings more evenly.	Thank you for your comment. This has been amended.
	Page: 14	this is verging on discussion as in results it presentation of the data with limited interpretation	Thank you for your comment. This has been amended
		Ahh here i can see some mapping being done	We thank the reviewer for this comment.

	Page: 15	The table assumes a level of familiarity with the coding system that not all readers may have. A table footnote explaining the coding would assist interpretation and improve readability.	We thank the reviewer for their valuable comment. a table footnote has been added
		What does this sentence add over what is in the table?	We thank the reviewer for this comment, and the sentence is now deleted.
	Page: 17	As the figure are not labelled in the attachment i canto assess the alignment to this section.	We thank the reviewer for this comment the figure number is now add as follows: "***Figure 3:*** *Framework types used throughout the design and redesign phases of HIRLs (Adapted from Goode et al., 2019)"*
		These are addressing a very similar topic.	We thank the reviewer for their observation, we have added the sentence accordingly<br><br>This scoping review aimed to identify, explore and mapped the TFs, applied philosophies and models underpinning HIRLs, the stages at which there applied and the rational for their use.
		If the prominence of particular theories is based solely on their identification in two studies rather than one, I would question the validity of this approach. As noted above, the difference between a theory appearing once versus twice is unlikely to represent a meaningful distinction in importance, influence, or relevance. Placing greater emphasis on selected theories on this basis risks overstating their prominence and may lead to unsupported conclusions. The authors should either provide a stronger justification for this approach or interpret the findings more cautiously.	We thank the reviewer for this comment this has been addressed.
		The coherence of this paragraph is poor. The opening sentence discusses the field being dominated by two specific theories; however, the discussion then shifts to the Hourglass model and subsequently to gaps in the literature. The relationship between these concepts is unclear, and there is insufficient linkage between ideas. As a result, the argument lacks a logical flow and is difficult to follow. The authors should consider	We thank the reviewer for their comment. We clarified this paragraph as shown on page number 18. The term 'hourglass' indicates that the patten of the findings are clustered around stages 1, 5 and 6 after we mapped it to Goode et al systems thinking.

		restructuring the paragraph to ensure that each concept is clearly connected and contributes to a coherent narrative.	
		It is unclear how the theories were classified and analysed. While the manuscript presents categories for frameworks, I could not identify a corresponding classification of theories according to their underlying foundations (e.g., behavioural, organisational, sociological, psychological, or implementation science). Without such categorisation, it is difficult to understand the theoretical basis of the included studies or compare how different theories were applied. The authors should clarify whether a formal classification was undertaken and, if so, describe the approach used. If not, consideration should be given to categorising theories according to their conceptual foundations to provide deeper insight into the findings.	We thank the reviewer for this comment. We have categorised the theories according to their conceptual foundations, adding a new column and footnote to Table 2, and have described this approach in the Methods as follows:<br><br>*"..Each theory was classified by its disciplinary origin, determined from the sources cited in the included studies (e.g. behavioural, organisational, sociological, psychological or implementation science). Data extraction and synthesis were conducted by AB and discussed with PB and COD. Any inconsistencies were resolved and agreed upon by the team…."*
	Page: 18	As supported by who?	Thank you for this comment. We have revised this section to improve clarity and have added supporting evidence, as follows:<br>*"…The application of Human Factors Engineering (HFE), systems thinking, and the Systems Engineering Initiative for Patient Safety (SEIPS) model is consistent with sociotechnical perspective on patient safety. 33,36. These frameworks conceptualise safety and performance as emerging form interactions among person, task, physical environment, technology and tools, organisation of work rather than just resulting from individual-level factors (Carayon et al., 2006; Holden et al., 2013). Their application supports a more holistic*

			approach to HIRL design and implementation, ensuring that requirements extend beyond user motivation. This includes system usability, accessibility, task alignment, and organisational contexts for example governance, feedback mechanises, resources, and healthcare employee capacity building to act on effective reporting incidents (Holden et al., 2013)….”
		There is no evidence to support these claims and as such they are opinion based and not grounded in the evidence.	Thank you for this comment. We have revised this section to improve clarity and have added supporting evidence, as follows: *“…The application of Human Factors Engineering (HFE), systems thinking, and the Systems Engineering Initiative for Patient Safety (SEIPS) model is consistent with sociotechnical perspective on patient safety. 33,36. These frameworks conceptualise safety and performance as emerging form interactions among person, task, physical environment, technology and tools, organisation of work rather than just resulting from individual-level factors (Carayon et al., 2006; Holden et al., 2013). Their application supports a more holistic approach to HIRL design and implementation, ensuring that requirements extend beyond user motivation. This includes system usability, accessibility, task alignment, and organisational contexts for example governance, feedback mechanises, resources, and healthcare employee capacity building to act on effective reporting incidents (Holden et al., 2013)..”*

		This has been discussed above already	We thank the reviewer for their comment. We clarified this paragraph as shown on page number 18. The term 'hourglass' indicates that the patten of the findings are clustered around stages 1, 5 and 6 after we mapped it to Goode et al systems thinking.
	Page: 19	The discussion lacks a clear interpretation of the findings and their implications for practice, research, or policy. Rather than synthesising the results and articulating their significance, this section reads largely as an alternative presentation of the findings. The authors should move beyond description to explain what the findings mean, why they matter, and what actions or recommendations arise from them. A stronger discussion of the implications for HIRL research and practice would strengthen the manuscript considerably.	We thank the reviewer for this insightful comment. We have clarified and revised the Discussion section and supported it with relevant evidence to make it clearer and more concise. Moreover, the section titled "Implications for practice and future research" in the original version has now been merged into the Discussion.
		This section reads more like an extension of the results than a discussion. It focuses on describing and classifying levels of integration rather than interpreting the findings. Greater emphasis is needed on what the findings mean, how they align with existing evidence, their implications for practice, and the actions or recommendations arising from them.	We thank the reviewer for this comment. We have revised this as follows:<br>1. Descriptive and classificatory statements have been moved to the results section (page 16).<br>2. For clarity, we have added information to the Methods to link the results and discussion (page 11).<br>3. We have revised the discussion and reframed the remaining text as an interpretation of the findings (page 19).
	Page: 31	This is repeated	We thank the reviewer for their observation the duplication now deleted.

	Page: 39	It is noted that the attention to capitalisation and punctuation is inconsistent in this table	We thank the reviewer for this comment this has been addressed
	Page: 43	What is this figure?	We thank the reviewer for this comment this has been amended
	Page: 45	There are no figure numbers to directly align these with the text, so it is difficult to assess which one goes were for certainty.	We thank the reviewer for this comment all figures are now labelled
		Is this Figure 3???	We thank the reviewer for this question. Yes, it is and now it has been labelled accordingly
	Page: 46	This is repeated but it is noted that the page numbers are not the same. Please ensure the correct checklist is provided	We thank the reviewer for this comment. Repeated table has been deleted, and page numbers are up to date. it will be modified accordantly
