# Supplementary material for: Theoretical frameworks shaping healthcare incident reporting and learning systems: a scoping review
Source: BMJ Open. 2026 Nov 9;16(11):e124497. doi: 10.1136/bmjopen-2026-124497 (PMC13756437; doi:10.1136/bmjopen-2026-124497)
Supplement: Supplementary file 2 [file bmjopen-16-11-s001.docx]

**Appendices:**

**Appendix 1:** PRISMA-ScR checklist for scoping review

**Appendix 2:** ECLIPSE criteria

**Appendix 3:** Search strategies table

**Appendix 4**: The included studies

**Appendix 5:** Characteristics of participants, reports, and reporting system in included studies **Appendix 6:** Classification of theoretical framework categories

Appendices:

Appendix One: PRISMA-ScR- Checklist for Scoping reivew

**Preferred Reporting Items for Systematic reviews and Meta-Analyses extension for Scoping Reviews (PRISMA-ScR) Checklist**

| **SECTION** | **ITEM** | **PRISMA-ScR CHECKLIST ITEM** | **REPORTED ON PAGE #** |
| --- | --- | --- | --- |
| **TITLE** | | | |
| Title | 1 | Identify the report as a scoping review. | Page no. 1 |
| **ABSTRACT** | | | |
| Structured summary | 2 | Provide a structured summary that includes (as applicable): background, objectives, eligibility criteria, sources of evidence, charting methods, results, and conclusions that relate to the review questions and objectives. | Page no. 2-4 |
| **INTRODUCTION** | | | |
| Rationale | 3 | Describe the rationale for the review in the context of what is already known. Explain why the review questions/objectives lend themselves to a scoping review approach. | Page no. 5-8 |
| Objectives | 4 | Provide an explicit statement of the questions and objectives being addressed with reference to their key elements (e.g., population or participants, concepts, and context) or other relevant key elements used to conceptualize the review questions and/or objectives. | Page no. 8 |
| **METHODS** | | | |
| Protocol and registration | 5 | Indicate whether a review protocol exists; state if and where it can be accessed (e.g., a Web address); and if available, provide registration information, including the registration number. | Page no. 8 |
| Eligibility criteria | 6 | Specify characteristics of the sources of evidence used as eligibility criteria (e.g., years considered, language, and publication status), and provide a rationale. | Page no. 9 |
| Information sources* | 7 | Describe all information sources in the search (e.g., databases with dates of coverage and contact with authors to identify additional sources), as well as the date the most recent search was executed. | Page no. 9 |
| Search | 8 | Present the full electronic search strategy for at least 1 database, including any limits used, such that it could be repeated. | Page no. 8 |
| Selection of sources of evidence† | 9 | State the process for selecting sources of evidence (i.e., screening and eligibility) included in the scoping review. | Page no. 9-10 |
| Data charting process‡ | 10 | Describe the methods of charting data from the included sources of evidence (e.g., calibrated forms or forms that have been tested by the team before their use, and whether data charting was done independently or in duplicate) and any processes for obtaining and confirming data from investigators. | Page no. 10 |
| Data items | 11 | List and define all variables for which data were sought and any assumptions and simplifications made. | Page no. 10 |
| Critical appraisal of individual sources of evidence§ | 12 | If done, provide a rationale for conducting a critical appraisal of included sources of evidence; describe the methods used and how this information was used in any data synthesis (if appropriate). | Page no. 10-11 |
| Synthesis of results | 13 | Describe the methods of handling and summarizing the data that were charted. | Page no. 10-11 |
| **RESULTS** | | | |
| Selection of sources of evidence | 14 | Give numbers of sources of evidence screened, assessed for eligibility, and included in the review, with reasons for exclusions at each stage, ideally using a flow diagram. | Page no. 12-17 |
| Characteristics of sources of evidence | 15 | For each source of evidence, present characteristics for which data were charted and provide the citations. | Page no. 12-17 |
| Critical appraisal within sources of evidence | 16 | If done, present data on critical appraisal of included sources of evidence (see item 12). | Page no. 11-17 |
| Results of individual sources of evidence | 17 | For each included source of evidence, present the relevant data that were charted that relate to the review questions and objectives. | Page no. 11-17 |
| Synthesis of results | 18 | Summarize and/or present the charting results as they relate to the review questions and objectives. | Page no. 11-17 |
| **DISCUSSION** | | | |
| Summary of evidence | 19 | Summarize the main results (including an overview of concepts, themes, and types of evidence available), link to the review questions and objectives, and consider the relevance to key groups. | Page no. 18-21 |
| Limitations | 20 | Discuss the limitations of the scoping review process. | Page no. 20-21 |
| Conclusions | 21 | Provide a general interpretation of the results with respect to the review questions and objectives, as well as potential implications and/or next steps. | Page no. 22 |
| **FUNDING** | | | |
| Funding | 22 | Describe sources of funding for the included sources of evidence, as well as sources of funding for the scoping review. Describe the role of the funders of the scoping review. | Page no. 22 |

JBI = Joanna Briggs Institute; PRISMA-ScR = Preferred Reporting Items for Systematic reviews and Meta-Analyses extension for Scoping Reviews.

* Where *sources of evidence* (see second footnote) are compiled from, such as bibliographic databases, social media platforms, and Web sites.

† A more inclusive/heterogeneous term used to account for the different types of evidence or data sources (e.g., quantitative and/or qualitative research, expert opinion, and policy documents) that may be eligible in a scoping review as opposed to only studies. This is not to be confused with *information sources* (see first footnote).

‡ The frameworks by Arksey and O’Malley (6) and Levac and colleagues (7) and the JBI guidance (4, 5) refer to the process of data extraction in a scoping review as data charting*.*

§ The process of systematically examining research evidence to assess its validity, results, and relevance before using it to inform a decision. This term is used for items 12 and 19 instead of "risk of bias" (which is more applicable to systematic reviews of interventions) to include and acknowledge the various sources of evidence that may be used in a scoping review (e.g., quantitative and/or qualitative research, expert opinion, and policy document).

*From:* Tricco AC, Lillie E, Zarin W, O'Brien KK, Colquhoun H, Levac D, et al. PRISMA Extension for Scoping Reviews (PRISMAScR): Checklist and Explanation. Ann Intern Med. 2018;169:467–473. [doi: 10.7326/M18-0850](http://annals.org/aim/fullarticle/2700389/prisma-extension-scoping-reviews-prisma-scr-checklist-explanation).

### Appendix Two: ECLIPSE criteria

| ECLIPSE element | Examples of Keywords |
| --- | --- |
| Expectation  What is the information you find going to be used to support? | Theoretical framework, human factors, ergonomics, theory |
| Client group  Who is the service for or elements of care is it addressing? | Patients in all health care settings  Reporting of errors or inclidents  Reporting by members of the healthcare workforce |
| Location  Where is the service run from? (e.g. community centre or hospital) | Hospital, clinic, primary healthcare |
| Impact  What is the desired change to service, if any?  How are you defining success?  How will you measure success? | Improving, reporting, enhancing reported, underreporting, incident reduction.  Use of theoretical framework, human factors approaches, ergonomics |
| Professionals  Who is providing the service?  Who will be involved in improving the service? | Healthcare worker from any healthcare profession |
| Service  Which service are you searching for information on? | Incident reporting, Learning systems, Patient safety events, Adverse events reporting, Near-miss events reporting, Sentinel events reporting, Never events, Underreporting |

### Appendix Three: Search Strategy

| Search strategy | |
| --- | --- |
| String A  (Theoretical framework) | (Theoretical adj framework*).mp. [mp=title, book title, abstract, original title, name of substance word, subject heading word, floating sub-heading word, keyword heading word, organism supplementary concept word, protocol supplementary concept word, rare disease supplementary concept word, unique identifier, synonyms, population supplementary concept word, anatomy supplementary concept word]  Or  (human adj factor*).mp. [mp=title, book title, abstract, original title, name of substance word, subject heading word, floating sub-heading word, keyword heading word, organism supplementary concept word, protocol supplementary concept word, rare disease supplementary concept word, unique identifier, synonyms, population supplementary concept word, anatomy supplementary concept word]  Or  exp "Theory of Planned Behavior"/ or exp *Information Theory/ or exp *"Theory of Mind"/ or Theory.mp. or exp *Psychological Theory/  Or  (((human adj factor*) or ergonomic*) adj3 (methodology or Approach or principle)).mp.  Or  (theor* adj3 (Framework* or Domain* or Approach*)).mp.  Or  (theor* adj3 (model* or process* or principle*)).mp.  Or  (Construct* adj3 (theor* or model* or process* or principle*)).mp.  Or  Theory of Planned Behavior.mp.  Or  exp "Theory of Planned Behavior"/  Or  (Normalization adj Process Theory).mp.  Or  (Socio-Technical adj Systems Theory).mp.  Or  (Organizational adj Learning Theory).mp.  Or  (High adj Reliability adj Organization Theory).mp.  Or  Behavioral Change Theory.mp.  Or  Generic analysis method.mp.  Or  Conceptual Framework for Incident Management.mp.  Or  (Normal adj Accident adj Theory).mp.  Or  Reason's accident causation model.mp. |
| String B  (patient safety events) | (sentinel adj event*).mp.  Or  patient safety incident*.mp.  Or  exp *Patient Harm/ or exp *Harm Reduction/ or harm*.mp.  Or  patient safety incident*.mp.  Or  (Prescrib* adj3 (event* or incident* or error*)).ti,ab,kw.  Or  exp *adverse drug event/ or exp *adverse drug reaction/ or Drug Safety.mp.  Or  exp *sentinel event*/ or exp *Risk Management/ or exp *Medical Errors/ or exp *Primary Health Care/ or exp *Safety Management/ or exp *Patient Safety/ or Patient safety incident.mp.  Or  (near adj miss*).mp. [mp=title, book title, abstract, original title, name of substance word, subject heading word, floating sub-heading word, keyword heading word, organism supplementary concept word, protocol supplementary concept word, rare disease supplementary concept word, unique identifier, synonyms, population supplementary concept word, anatomy supplementary concept word]  Or  (adverse adj event*).mp. [mp=title, book title, abstract, original title, name of substance word, subject heading word, floating sub-heading word, keyword heading word, organism supplementary concept word, protocol supplementary concept word, rare disease supplementary concept word, unique identifier, synonyms, population supplementary concept word, anatomy supplementary concept word]  Or  ((never adj event*).mp. [mp=title, book title, abstract, original title, name of substance word, subject heading word, floating sub-heading word, keyword heading word, organism supplementary concept word, protocol supplementary concept word, rare disease supplementary concept word, unique identifier, synonyms, population supplementary concept word, anatomy supplementary concept word]  Or  (medical adj mistake*).mp. [mp=title, book title, abstract, original title, name of substance word, subject heading word, floating sub-heading word, keyword heading word, organism supplementary concept word, protocol supplementary concept word, rare disease supplementary concept word, unique identifier, synonyms, population supplementary concept word, anatomy supplementary concept word]  Or  exp *Diagnostic Errors/ or exp *Medical Errors/ or exp *Medication Errors/ or (error* or mistake*).mp.  Or  ((exp health*/ or care*/) adj (worker or employee or staff or provider).mp.) and ((negligen* or malpractice*).mp. or Medical Error*/) [mp=title, book title, abstract, original title, name of substance word, subject heading word, floating sub-heading word, keyword heading word, organism supplementary concept word, protocol supplementary concept word, rare disease supplementary concept word, unique identifier, synonyms, population supplementary concept word, anatomy supplementary concept word]  Or  (exp Physicians/ or Surgeons/) and ((negligen* or malpractice*).mp. or Medical Error*/)  Or  (exp nurse/ or midwife/) and ((negligen* or malpractice*).mp. or Medical Error*/) [mp=title, book title, abstract, original title, name of substance word, subject heading word, floating sub-heading word, keyword heading word, organism supplementary concept word, protocol supplementary concept word, rare disease supplementary concept word, unique identifier, synonyms, population supplementary concept word, anatomy supplementary concept word]  Or  exp *Failure to Rescue, Health Care/ or exp *Injury Severity Score/ or exp *Hospital Mortality/ or failure*.mp. or injur*.mp. [mp=title, book title, abstract, original title, name of substance word, subject heading word, floating sub-heading word, keyword heading word, organism supplementary concept word, protocol supplementary concept word, rare disease supplementary concept word, unique identifier, synonyms, population supplementary concept word, anatomy supplementary concept word]  Or  (critical adj incident*).mp. [mp=title, book title, abstract, original title, name of substance word, subject heading word, floating sub-heading word, keyword heading word, organism supplementary concept word, protocol supplementary concept word, rare disease supplementary concept word, unique identifier, synonyms, population supplementary concept word, anatomy supplementary concept word]  Or  (sentinel adj (event* or incident* or harm* or accident*)).mp. [mp=title, book title, abstract, original title, name of substance word, subject heading word, floating sub-heading word, keyword heading word, organism supplementary concept word, protocol supplementary concept word, rare disease supplementary concept word, unique identifier, synonyms, population supplementary concept word, anatomy supplementary concept word]  Or  (medical adj error*).mp. [mp=title, book title, abstract, original title, name of substance word, subject heading word, floating sub-heading word, keyword heading word, organism supplementary concept word, protocol supplementary concept word, rare disease supplementary concept word, unique identifier, synonyms, population supplementary concept word, anatomy supplementary concept word]  Or  (patient adj safety).mp. [mp=title, book title, abstract, original title, name of substance word, subject heading word, floating sub-heading word, keyword heading word, organism supplementary concept word, protocol supplementary concept word, rare disease supplementary concept word, unique identifier, synonyms, population supplementary concept word, anatomy supplementary concept word]  Or  (sentinel adj event*).mp. [mp=title, book title, abstract, original title, name of substance word, subject heading word, floating sub-heading word, keyword heading word, organism supplementary concept word, protocol supplementary concept word, rare disease supplementary concept word, unique identifier, synonyms, population supplementary concept word, anatomy supplementary concept word]  Or  incident*.mp. or exp *Mass Casualty Incidents/ or exp *Risk Factors/  Or  exp *Patient Harm/ or exp *Harm Reduction/ or harm*.mp.  Or  (adverse adj2 (interaction$ or response$ or effect$ or event$ or reaction$ or outcome$)).ti,ab,kw.  Or  (exp report*/ or underreport*/) and (side effect* or complication* or toxicity or pharmacovigilance).mp. and ((error* or event*).mp. or incident*/) [mp=title, book title, abstract, original title, name of substance word, subject heading word, floating sub-heading word, keyword heading word, organism supplementary concept word, protocol supplementary concept word, rare disease supplementary concept word, unique identifier, synonyms, population supplementary concept word, anatomy supplementary concept word]  Or  (unintended adj harm).mp. [mp=title, book title, abstract, original title, name of substance word, subject heading word, floating sub-heading word, keyword heading word, organism supplementary concept word, protocol supplementary concept word, rare disease supplementary concept word, unique identifier, synonyms, population supplementary concept word, anatomy supplementary concept word]  Or  (unwanted adj harm).mp. [mp=title, book title, abstract, original title, name of substance word, subject heading word, floating sub-heading word, keyword heading word, organism supplementary concept word, protocol supplementary concept word, rare disease supplementary concept word, unique identifier, synonyms, population supplementary concept word, anatomy supplementary concept word]  Or  (unexpected adj harm).mp. [mp=title, book title, abstract, original title, name of substance word, subject heading word, floating sub-heading word, keyword heading word, organism supplementary concept word, protocol supplementary concept word, rare disease supplementary concept word, unique identifier, synonyms, population supplementary concept word, anatomy supplementary concept word]  Or  (unwanted adj harm).mp. [mp=title, book title, abstract, original title, name of substance word, subject heading word, floating sub-heading word, keyword heading word, organism supplementary concept word, protocol supplementary concept word, rare disease supplementary concept word, unique identifier, synonyms, population supplementary concept word, anatomy supplementary concept word]  Or  (unexpected adj harm).mp. [mp=title, book title, abstract, original title, name of substance word, subject heading word, floating sub-heading word, keyword heading word, organism supplementary concept word, protocol supplementary concept word, rare disease supplementary concept word, unique identifier, synonyms, population supplementary concept word, anatomy supplementary concept word]  Or  (undesirable adj harm).mp. [mp=title, book title, abstract, original title, name of substance word, subject heading word, floating sub-heading word, keyword heading word, organism supplementary concept word, protocol supplementary concept word, rare disease supplementary concept word, unique identifier, synonyms, population supplementary concept word, anatomy supplementary concept word]  Or  (drug safety adj3 (event* or incident* or error*)).ti,ab,kw.  Or  (drug surveillance or ((postmarketing or post marketing) adj2 surveillance)).ti,ab,kw.  Or  product surveillance.ti,ab,kw.  Or  drug monitoring.ti,ab,kw.  Or  (harm or harms or harmful).ti,ab,kw.  Or  (prevent or preventable or prevention*).ti,ab,kw.  Or  treatment emergent.ti,ab,kw.  Or  (iatrogenic or iatrogenesis).ti,ab,kw.  Or  (drug withdrawal* adj3 (event* or incident* or error*)).ti,ab,kw.  Or  ((Product Surveillance or Postmarketing) adj3 (error* or event* or incident*)).mp.  Or  (Adverse Drug Reaction Reporting System* adj3 (error* or event* or incident*)).mp.  Or  (Medication adj3 (event* or incident* or error*)).ti,ab,kw.  Or  (Drug Monitoring adj3 (system* or approach* or mechanism or method*)).ti,ab,kw.  Or  exp "Drug-Related Side Effects and Adverse Reactions"/  Or  Abnormalities, Drug-Induced/  Or  exp Postoperative Complications/ adj3 (error* or event* or incident*).mp.  Or  exp Intraoperative Complications/ adj3 (error* or event* or incident*).mp.  Or  exp Safety-Based Drug Withdrawals/ adj3 (error* or event* or incident*).mp.  Or  sentinal event*.mp.  Or  patient safety event*.mp.  Or  (critical adj event*).mp.  Or  exp *Medical Errors/ or exp *Near Miss, Healthcare/ or near miss.mp. or exp *Patient Safety/never event.mp. or exp *Medical Errors/  exp *Patient Harm/ |
| String C  (Incident reporting systems) | (sentinel adj event*).mp.  Or  exp *Medical Errors/ or exp *Quality Assurance, Health Care/ or exp *Accidental Falls/ or exp *Patient Safety/ or exp *Medication Errors/ or exp *Risk Management/ or exp *Humans/ or Incident reporting system.mp. or exp *Anesthesia/  (risk adj management).mp. [mp=title, book title, abstract, original title, name of substance word, subject heading word, floating sub-heading word, keyword heading word, organism supplementary concept word, protocol supplementary concept word, rare disease supplementary concept word, unique identifier, synonyms, population supplementary concept word, anatomy supplementary concept word]  Or  Direct patient* report* error* event* incident*.mp.  Or  Selfreport* error* event* incident*.mp.  Or  Under* report* error* event* incident*.mp.  Or  volunteer* report* error* event* incident*.mp.  Or  (incident adj reporting adj system*).mp. [mp=title, book title, abstract, original title, name of substance word, subject heading word, floating sub-heading word, keyword heading word, organism supplementary concept word, protocol supplementary concept word, rare disease supplementary concept word, unique identifier, synonyms, population supplementary concept word, anatomy supplementary concept word]  Or  (error adj report*).mp. [mp=title, book title, abstract, original title, name of substance word, subject heading word, floating sub-heading word, keyword heading word, organism supplementary concept word, protocol supplementary concept word, rare disease supplementary concept word, unique identifier, synonyms, population supplementary concept word, anatomy supplementary concept word]  (critical adj incident adj technique).mp. [mp=title, book title, abstract, original title, name of substance word, subject heading word, floating sub-heading word, keyword heading word, organism supplementary concept word, protocol supplementary concept word, rare disease supplementary concept word, unique identifier, synonyms, population supplementary concept word, anatomy supplementary concept word]  (safety adj report*).mp. [mp=title, book title, abstract, original title, name of substance word, subject heading word, floating sub-heading word, keyword heading word, organism supplementary concept word, protocol supplementary concept word, rare disease supplementary concept word, unique identifier, synonyms, population supplementary concept word, anatomy supplementary concept word]  (incident adj report*).mp. [mp=title, book title, abstract, original title, name of substance word, subject heading word, floating sub-heading word, keyword heading word, organism supplementary concept word, protocol supplementary concept word, rare disease supplementary concept word, unique identifier, synonyms, population supplementary concept word, anatomy supplementary concept word]  (reporting adj system).mp. [mp=title, book title, abstract, original title, name of substance word, subject heading word, floating sub-heading word, keyword heading word, organism supplementary concept word, protocol supplementary concept word, rare disease supplementary concept word, unique identifier, synonyms, population supplementary concept word, anatomy supplementary concept word]  (national adj reporting adj2 learning adj system).mp. [mp=title, book title, abstract, original title, name of substance word, subject heading word, floating sub-heading word, keyword heading word, organism supplementary concept word, protocol supplementary concept word, rare disease supplementary concept word, unique identifier, synonyms, population supplementary concept word, anatomy supplementary concept word]  (Information adj Technology adj Infrastructure adj Library).mp.  patient/ adj safety.mp. adj system.mp. adj failure*.mp. [mp=title, book title, abstract, original title, name of substance word, subject heading word, floating sub-heading word, keyword heading word, organism supplementary concept word, protocol supplementary concept word, rare disease supplementary concept word, unique identifier, synonyms, population supplementary concept word, anatomy supplementary concept word]  (Developing adj Incident adj Management adj Systems).mp.  implement*/ adj Incident.mp. adj learn*.mp. adj Management.mp. adj System*.mp. [mp=title, book title, abstract, original title, name of substance word, subject heading word, floating sub-heading word, keyword heading word, organism supplementary concept word, protocol supplementary concept word, rare disease supplementary concept word, unique identifier, synonyms, population supplementary concept word, anatomy supplementary concept word]  Design*/ adj Incident.mp. adj learn*.mp. adj Management.mp. adj System*.mp. [mp=title, book title, abstract, original title, name of substance word, subject heading word, floating sub-heading word, keyword heading word, organism supplementary concept word, protocol supplementary concept word, rare disease supplementary concept word, unique identifier, synonyms, population supplementary concept word, anatomy supplementary concept word]  (function* adj Incident adj learn* adj Management adj System*).mp. [mp=title, book title, abstract, original title, name of substance word, subject heading word, floating sub-heading word, keyword heading word, organism supplementary concept word, protocol supplementary concept word, rare disease supplementary concept word, unique identifier, synonyms, population supplementary concept word, anatomy supplementary concept word]  (investigate* adj system).mp.  (Geographic adj Information adj System Technology).mp.  (Information adj Technology adj Infrastructure adj system).mp.  Incident Command System.mp.  (Error adj report* adj system).mp.  (health* adj3 (incident report* or learn* or system)).mp.  (report* adj learn* adj system).mp. |
| Google Scholar | ("incident reporting" OR "patient safety events" OR "learning system") AND (theory OR framework OR model) |

**Appendix four: The included studies**

|  | **Author(s)** | **Year** | **Country** | **Setting** | **Type of reporter (participant)** | **Number of participants** | **Theoretical Framework, applied philosophies and models Type** | **Framework categories** | **Application Stage** | **Level of Integration** |
| --- | --- | --- | --- | --- | --- | --- | --- | --- | --- | --- |
| 1 | Abry,S., et al | 2022 | Iran | 8 public hospitals | Nurses | 326 participants | Theory of Planned Behaviour (TPB) | Predictive & Evaluative | Evaluation | Partial |
| 2 | Al-Rayes,S., et al | 2020 | Sudia Arabia | The Dental Hospital | The participants included faculty, clinical and non-clinical staff, | 201 participants | Technology Acceptance Model (TAM) | Predictive | Design | Superficial |
| 3 | Alqubaisi,M., et al | 2016 | UAE | 3 major medical/ surgical hospitals | The participants were health professionals, specifically nurses, pharmacists, and physicians. | 29 participants | Theoretical Domains Framework (TDF) | Explanatory | Design | Partial |
| 4 | Archer, S., et al | 2017 | UK | Healthcare settings, | The participants in this study were healthcare professionals (HCPs) | 29726 participants. | Framework derived using a grounded theory approach | Explanatory | Multi Stage | Partial |
| 5 | Armitage, et al | 2009 | UK | NHS teaching hospital | The participants in the study were healthcare professionals (Doctors, Nurses and Pharmacists | 40 participants. | Human Factors Engineering (HFE) | Explanatory | Evaluation | Fully |
| 6 | Berdahl, et al | 2023 | USA | Hospital setting, | The study was conducted in a hospital setting, specifically Cedars-Sinai Medical Center, a hybrid academic/community hospital | 1980 participants | Human Factors Engineering (HFE) and the Consolidated Framework for Implementation Research (CFIR) | Explanatory | Evaluation | Partial |
| 7 | de Kam, et al | 2020 | Netherlands | Dutch hospitals reported and investigated 4667 incidents. | Participants included healthcare professionals, incident investigators, quality managers, chairs of investigation committees, healthcare inspectors, and other | 73 semi-structured interviews and 36 hours of ethnographic observations | The conceptual framework of social and participative learning.and incident investigation processes. | Evaluative | Multi Stage | Fully |
| 8 | de la Torre-Perez, et al | 2023 | Spain | A high-complexity reference hospital | Incidents/Healthcare professionals, including doctors, nurses, assistant nurses, medical residents, pharmacists, administrative staff, and others | 6,379 incident notifications were reported during the study period (2016–2019). | The conceptual framework for patient safety and incident reporting systems (IRSs) influenced by frameworks: 1. Safety Culture Framework 2. Human Factors Framework 3. WHO Conceptual Framework | Evaluative | Not specify | Partial |
| 9 | Ferroli, et al | 2012 | Italy | Neurosurgical department within a hospital setting, | Near-miss events/Participants included all members of the neurosurgical department, such as surgeons, anaesthesiologists, and other operating room staff | 14 near-miss events reported | Reason Swiss-Cheese model and the Heinrich Pyramid model | Explanatory | Multi Stage | Fully |
| 10 | Gong, Y. | 2010 | USA | Hospital setting, specifically the University of Missouri Health Care System (UMHC), which includes 5 facilities located in mid-Missouri. | Incidents/ healthcare professionals who reported incidents in the voluntary medical incident reporting system. The majority were registered nurses (66.2%), with other professionals such as unit clerks and physical therapists contributing 5.0% of the reports. | 2,919 voluntary incident. | Goals, Operators (GOMS) model, theory of Human-Computer Interaction (HCI).and the Keystroke-Level Model (KLM), | Predictive & Evaluative | Evaluation | Partial |
| 11 | Gong, Y. | 2011 | USA | Hospital setting, | Incident reports/Participants included healthcare professionals such as registered nurses, anonymous reporters, pharmacists, physicians, respiratory therapists, lab technicians, managers, and others involved in reporting incidents. | The study analyzed a total of 2,919 unique voluntary incident reports. | Human Factors Engineering (HFE) and ontology-based system design | Evaluative | Evaluation | Partial |
| 12 | Hartnell, et al | 2012 | Canada | 4 community hospitals | Participants included front-line healthcare | 27 participants across the focus groups | Safety Culture Theory | Explanatory | Multi Stage | Fully |
| 13 | Jegathesan, et al | 2016 | USA | Children’s Hospital | Participants included paediatric residents, fellows, and attending physicians from the Department of Paediatrics. | 349 individuals, and 176 completed questions, representing a 50% response rate. | Carol Dweck’s Mindset Theory as its theoretical framework, | Explanatory | Design | Fully |
| 14 | Lacson, et al | 2019 | USA | Hospital setting, | Patient safety events related to diagnostic imaging were reported in the ESRS.and healthcare providers who submitted reports indirectly contributed to the study. | 854 report (out of 11,570 total safety reports submitted to the ESRS in 2015). | Systems Engineering Initiative for Patient Safety (SEIPS) model | Descriptive & explanatory | Evaluation | Fully |
| 15 | Lee, et al | 2024 | Taiwan | Hospital setting, | Participants included healthcare staff such as doctors, nurses, medical technicians, and administrators. | 626 participant (423 in the preintervention group and 203 in the postintervention group). | TRIZ-based model (Theory of Inventive Problem Solving), integrated with the SERVQUAL methodology for service quality assessment. | Evaluative | Multi Stage | Fully |
| 16 | Liang, C. and Gong, Y. | 2015 | USA | The study examines patient safety event reporting systems, | The participants in the study are patient safety event reports, which are categorized into two subcategories: "device-related complications" and "diagnostic errors." | 110 reported events | K-nearest neighbour (KNN) | Predictive | Implementation | Fully |
| 17 | MacHen, S. | 2023 | UK | Hospital setting, | The study involves a multidisciplinary team with varying levels of knowledge of safety science working in an acute healthcare setting. | The thematic review template was trialled with a review of six reference cases. | Systems thinking theoretical framework, operationalised by the Contributory and Mitigations Factors Classification | Descriptive | Multi Stage | Fully |
| 18 | Morag, et al | 2012 | Multi countries | 2 tertiary care hospitals | Incidents report/ Participants included clinical staff members | 359 incidents reports | Human Factors Engineering (HFE) | Descriptive & evaluative | Multi Stage | Fully |
| 19 | Noble, et al | 2011 | USA | The study examines healthcare settings at local and national levels | The participants are healthcare organisations and systems, including hospitals, regional practices, and national reporting systems. Individual healthcare workers are involved in reporting incidents within these systems. | Six patient safety reporting systems. | The study uses a framework based on public health principles and classifications provided by the World Health Organization (WHO). | Evaluative | Evaluation | Partial |
| 20 | Oyibo, et al | 2024 | Canada | The community pharmacy sector. | Pharmacy professionals/stakeholders with experience in MIRLS | 3 expert system evaluators | Persuasive System Design (PSD) taxonomy. | Evaluative | Multi Stage | Partial |
| 21 | Pham, et al | 2010 | UK | The study examined patient safety reporting systems (PSRS) | The participants were incident reports submitted by healthcare providers to the NRLS, rather than individual human participants. | 104,674 incident reports from 20 trusts and 12 distinct work areas were analysed. | Risk Resiliency Concept as the theoretical framework, adapted from the aviation industry to develop the Harm Susceptibility Model (HSM). | Evaluative | Evaluation | Fully |
| 22 | Jabin, et al. | 2023 | Sweden | Hospital setting, | The participants indirectly involved were healthcare professionals who reported incidents through digital systems. The study analysed their reports and the manufacturers' conclusions. | 38 incident reports were collected, but only 37 reports were analyzed after excluding one non-HIT-related incident. | Health Information Technology Classification System (HIT-CS) | Evaluative | Evaluation | Fully |
| 23 | Stewart, et al | 2018 | Qatar | Secondary and tertiary care settings. | The participants were healthcare professionals, including doctors, nurses, and pharmacists working within HMC. | 1,604 healthcare professionals completed the survey, and 54 healthcare professionals participated in the focus groups. | Theoretical Domains Framework (TDF) | Explanatory | Design | Fully |
| 24 | Williams et al., | 2015 | UK | Hospital setting, | Hospital pharmacists | 270 hospital pharmacists (response rate = 45%). | Theory of Planned Behaviour (TPB) | Explanatory | Design | Fully |
| 25 | Lawton, et al | 2012 | Multi countries | Hospital setting, | Participants included healthcare staff (e.g., nurses, doctors, pharmacists, anaesthetists), patients, and human factors experts. | The systematic review included 95 studies representing 83 independent datasets, with a total of 1676 contributory factors extracted | Reason Swiss-Cheese model | Descriptive and Explanatory | Design | Fully |
| 26 | Holmström, et al | 2012 | Multi countries | The study examined both national and local healthcare settings. | National medication safety experts knowledgeable about medication error reporting systems in their respective countries | 16 medication safety experts from 16 different countries | Human Factors Engineering (HFE) and a systems approach | Explanatory | Design | Fully |
| 27 | Chang and Hsu | 2012 | Taiwan | National healthcare setting, specifically medical centers and regional hospitals | Medical staff, including physicians, nurses, pharmacists, medical lab technologists, and radiologists. | 450 questionnaires were distributed, 331 were returned, and 183 valid samples were used for analysis. | Unified Theory of Acceptance and Use of Technology (UTAUT), which was modified by integrating the Value of Perceived Consequence (VPC) | Predictive | Evaluation | Fully |
| 28 | Stavropoulou,et al | 2015 | Multi countries | The study examined various healthcare settings, including general hospitals, specialized units | Participants in the reviewed studies included healthcare practitioners such as doctors, nurses, allied health professionals, and other clinical staff. | The systematic review analysed a total of 43 studies | Argyris and Schön’s theory of organisational learning, specifically focusing on the concepts of single-loop and double-loop learning. | Evaluative | Evaluation | Partial |

**Appendix Five:** Characteristics of participants, reports, and reporting system in included studies.

| Characteristics of participants | | Number of included studies | Study citation |
| --- | --- | --- | --- |
| Incidents reports | The reports | 8 | 30,31,34,38,49,50,51,52 |
| Nurses, pharmacists, and physicians and other healthcare staff. | The reporter | 5 | 6,40,42,43,55 |
| Nurses, pharmacists, and physicians. |  | 4 | 33,36,46,47 |
| Nurses |  | 2 | 29,48 |
| Nurses, pharmacists, physicians and anaesthetists, patients, and human factors experts. |  | 1 | 54 |
| Participants included Pediatric residents, fellows, and attending physicians from the Department of Pediatrics. |  | 1 | 32 |
| Medication safety experts |  | 1 | 53 |
| Pharmacists |  | 1 | 39 |
| Pharmacy /stakeholders with experience in MIRLS |  | 1 | 41 |
| Students, interns, dentists, nurses/dental assistants, administrative staff, and others |  | 1 | 45 |
| Healthcare professionals, incident investigators, quality managers, chairs of investigation committees, healthcare inspectors, and other stakeholders. |  | 1 | 44 |
| Multidisciplinary team |  | 1 | 37 |
| Incidents reporting systems. | The reporting system | 1 | 35 |

**Appendix Six:** classification of theoretical framework categories
